# Lipid Profile of Children with Malaria by* Plasmodium vivax*

**DOI:** 10.1155/2016/9052612

**Published:** 2016-12-05

**Authors:** Rosa Maria Dias, Jose Luiz Fernandes Vieira, Bianca da Conceição Cabral, Isameriliam Rosaulem Pereira da Silva, Laelia Maria Barra Feio Brasil, Eliete da Cunha Araújo, Marcieni Ataíde de Andrade

**Affiliations:** Health Science Institute, Pará Federal University, Belém, PA, Brazil

## Abstract

*Background*. Changes in lipid profile are commonly reported in adult patients with malaria. However, a few studies evaluated lipid abnormalities in children continuously exposed to* P. vivax*.* Objective*. To evaluate lipid abnormalities in children with* P. vivax* infection and to assess if parasite count or the history of malaria correlates with lipid levels at admission.* Methods*. A total of 75 children were included in the study, from which 43 were slide confirmed infection by* P. vivax*. Serial blood samples were collected at admission and, on days 7 and 14, evaluated for the colorimetric measurements of triglycerides, very low-density lipoprotein (VLDL), total cholesterol, high-density lipoprotein (HDL), and low-density lipoprotein (LDL).* Results*. The levels of total cholesterol, LDL, and HDL were significantly lower in malaria cases. The levels of VLDL and triglycerides were significantly higher in children with malaria. Such changes were transient and were not associated with parasite counting as well as with the history of malaria of patients.* Conclusion*. There are significant lipid abnormalities in children with low level of* P. vivax* infection and mild signs and symptoms of the disease, which are not associated with parasitaemia and previous episodes of disease.

## 1. Introduction

Malaria is a major public health issue in several tropical countries [1]. In Brazil, approximately 131,000 cases occur each year, most of them in the Amazon basin.* Plasmodium vivax* is the most prevalent species and accounts for 85% of the cases [1, 2]. Most of the cases are characterized by a low parasite density with mild or even absent signs and symptoms of the disease [2–5]. However, several laboratorial abnormalities occur in the course of the infection, such as anemia, thrombocytopenia, methemoglobinemia, and lipid blood levels [6, 7].

Changes in serum lipid profile have been associated with genetics and with infectious and inflammatory diseases as well as in chronic or acute conditions [8, 9]. In malaria, a recent meta-analysis study has proved that the changes in serum lipids are a characteristic feature of the disease [10]. Patients with malaria present hypocholesterolemia, decreased levels of high-density lipoproteins (HDL) and low-density lipoproteins (LDL), which are accompanied by increased levels of triglycerides, and very low-density lipoproteins (VLDL). Such lipid abnormalities are transient, occurring in the most prevalent species of* Plasmodium* as well as in complicated or noncomplicated cases [10–13].

Most of the studies designated to evaluate lipid abnormalities were performed in adult patients with* P. falciparum* [11, 14, 15]. However, in most of the endemic areas, including the Brazilian Amazon basin,* P. vivax* is the most prevalent species and children have a high incidence of the disease. There is a paucity of data describing lipid abnormalities in children with vivax malaria. Such changes can increase the risk of chronic diseases, as atherosclerosis and related diseases, which is relevant for children continuously exposed to malaria [14, 16, 17]. Additionally, data correlating the parasitaemia at admission or the history of malaria with lipid levels are inconclusive in children [10]. Thus, the aim of the current study was to evaluate the lipid profile of children with low-level* P. vivax* infection and to assess if parasite count and history of malaria correlate with lipid levels at admission.

## 2. Population and Methods

### 2.1. Ethical Statement

The study was submitted to Plataforma Brasil under protocol CAAE 2 07199612.0.0000.0018 and approved under the number 261.593/2013.

### 2.2. Study Site and Participants

The study was carried out in the municipality of Anajas in the Marajo Island (00° 59′ 21′′S and 49° 56′ 24′′W). Anajas has an area of 6,912 Km^2^ and 27,540 inhabitants, from which 7,347 are children aged 2–10. The economy is based on agriculture practices, timber extraction, and fishing. The municipality accounts for 17.4% of malaria cases in the state of Pará and has a constant annual incidence of cases above 50/1,000 inhabitants. Most cases (86.3%) are caused by* P. vivax*. The municipality has seven health public facilities, including an ambulatory for the diagnosis and treatment of malaria.

Participants were recruited by spontaneous demand among those who referred for attendance at health facilities of the municipality from January to December, 2014. Two groups of children were randomly included in the study: (1) children with slide confirmed infection by* P. vivax* and (2) children with negative diagnoses of malaria after microscopic examination of three blood smears. Exclusion criteria included patients with mixed malaria or signs and symptoms of severe malaria (jaundice, renal or pulmonary impairment, severe anemia, and altered level of consciousness), chronic and other parasitic diseases, and obesity and underweight assessed by the body mass index and those who have reported a history of malaria infection within the previous two months.

### 2.3. Treatment and Follow-Up

Children with* P. vivax* were treated with chloroquine and primaquine according to the recommendation of the World Health Organization [18]. Doses of antimalarial drugs were adjusted by the weight of the patients. The community health workers were responsible for prescribing and dispensing the drugs under the supervision of the medical staff. The guardians of children received specific instructions about the administration on each day and were strongly advised to complete the course of treatment. Social-demographic and clinical questionnaires were applied to all guardians of children before their enrollment in the study. Data regarding to the fever and the history of malaria, including the last episode of the disease as well as the use of antimalarial drugs, were carefully recorded. Thereafter, patients of both groups were classified as no reports of malaria; 1–3 episodes; or 4 or more episodes of the disease in their life. All patients were requested to return for follow-up on days 1, 2, 3, 4, 7, and 14 or at any time whenever signs and symptoms suggestive of malaria occur. A parasite count was performed at each visit.

### 2.4. Blood Sampling and Laboratory Analysis

Serial blood samples were collected at baseline (D0) in both groups of children recruited for the study and on days 7 and 14 after the commencement of the treatment in children with vivax malaria. Samples were collected after a fasting of 12 h. Triglycerides, total cholesterol, HDL, and LDL were measured by spectrometer methods using Merck- diagnosis kits, following the good laboratory practices. VLDL was measured by enzyme-linked immunosorbent assay (LSBio Inc.). Samples were analyzed in triplicate. The normal ranges of each lipid followed the recommendations of the Brazilian Cardiology Society [19].

### 2.5. Microscopic Examination

Parasite counts were performed every 24 hours in Giemsa-stained thick films until clearance. Blood films were examined microscopically using 100x (oil immersion) objectives. Parasite density was expressed as the number of parasites per microliter of blood. This was derived from the number of parasites per 200 white blood cells in a thick film, considering a total white blood cell count of 8,000. The limit of detection of parasites was 40/*µ*L [20].

### 2.6. Data Analysis

Data are presented as a mean and standard deviation and, if necessary, as median and range. Chi-square test was used to compare proportions of altered lipid levels between groups of study. Mann–Whitney *U* test was used to compare the lipids levels between groups at admission on the study. A univariate analysis was used to compare lipid levels amongst days of blood sampling in children with malaria as well as to compare lipid levels amongst patients classified according to the number of previous episodes of malaria. Spearman rank correlation was used to estimate the correlations between parasitaemia at admission with triglycerides, total cholesterol, and fractions. Data were analyzed using SPSS software, Release 21 (IBMinc, Chicago, IL, USA). The significance level accepted was 5%.

## 3. Results

A total of 75 children met the criteria for their inclusion in the study. Children with positive slide confirmed infection by* P. vivax* corresponded to 57.3% (*n* = 43) of the participants. The baseline characteristics of participants are shown in Table 1.

**Figure 1:**
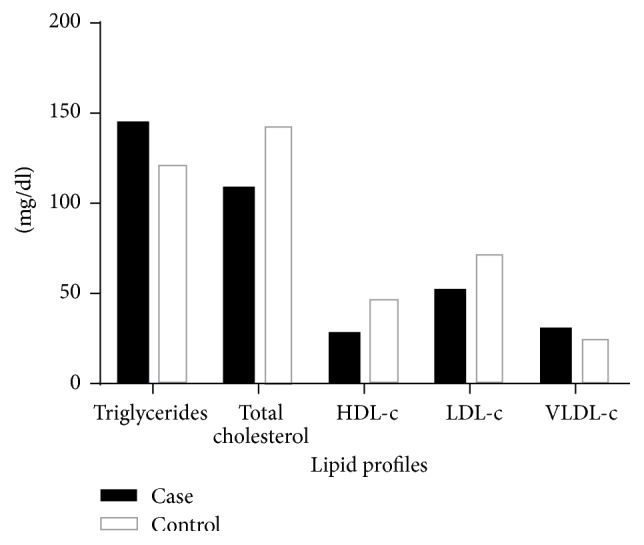
Lipid profile of children with vivax malaria (■) and control group (□).

At admission, triglycerides levels were higher in patients with malaria (ranges: 71 to 196 mg/dl) when compared to healthy children (68–243 mg/dl) (*U* = 403.5; *p* = 0.0011). A similar result was found for VLDL fraction (*U* = 338; *p* = 0.003), with the higher values in malaria cases (15.5–68.7 mg/dl), compared to healthy children (15–39.2 mg/dl). The total cholesterol was significantly lower (*U* = 338.5; *p* < 0.001) in malaria cases (82–208 mg/dl) compared to healthy children (ranges: 97–220 mg/dl). As expected, HDL levels were significantly lower (*U* = 122; *p* < 0.001) in malaria cases (ranges: 2–49 mg/dl) compared to healthy children (ranges: 32–61 mg/dl). The values of LDL (*p* < 0.001) were also lower (*U* = 441; *p* < 0.001) in children with malaria (9–136 mg/dl), compared to healthy children (14–138 mg/dl). The lipid levels comparison between case and control subjects is shown in Figure 1.

The proportion of patients with triglycerides above normal ranges was also higher in malaria cases (55.8%) when compared to control group (25%) (*χ* ^2^ = 11.74; *p* = 0.0006). For total cholesterol, there are no values below the normal ranges in both groups. Additionally, the proportion of malaria cases with values of HDL below normal ranges (92.8%) was significantly higher than the control group (40.62%) (*χ* ^2^ = 20.83; *p* < 0.0001). Only children with malaria (97.6%) showed LDL levels below normal range. In the control group, most children (93.7%) showed LDL levels within the normal range and there were no values below normal range.

**Figure 2:**
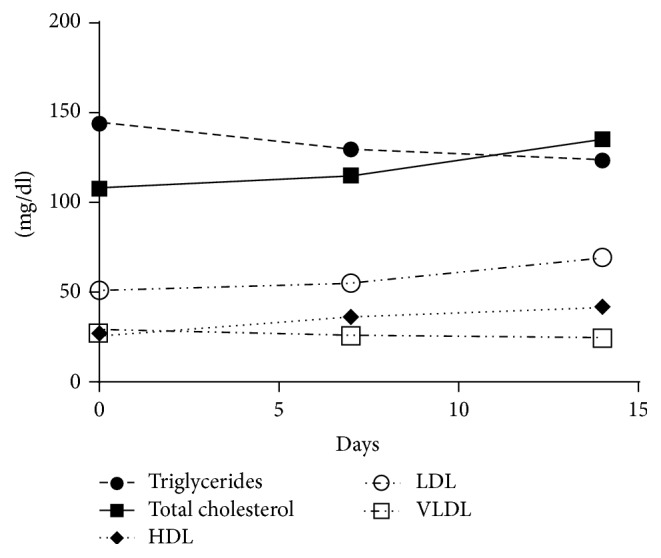
Temporal evaluation of lipid profiles of children with vivax malaria.

Significant changes in serum lipid profiles were seen in the follow-up, as follows: triglycerides (*F* = 3.1485; *p* = 0.046) and VLDL (*F* = 6.972; *p* = 0.0019) decreased significantly between D0 and D14. On the other hand, total cholesterol (*F* = 16.78; *p* < 0.001), HDL (*F* = 16.69; *p* < 0.001), and LDL (*F* = 9.414; *p* < 0.01) increased significantly between D0 and D14. The evolution of lipid concentrations versus time is shown in Figure 2.

The number of previous episodes of malaria was not associated with triglycerides (*F* = 0.5249; *p* = 0.599), total cholesterol (*F* = 0.7244; *p* = 0.504), HDL (*F* = 0.2820; *p* = 0.7595), LDL (*F* = 1.2768; *p* = 0.2896), and VLDL (*F* = 0.7259; *p* = 0.5056).

Finally, there were no significant correlations between the parasitaemia at admission with triglycerides (*rs* = −0.126; *p* = 0.3305), total cholesterol (*rs* = 0.0269; *p* = 0.8370), HDL (*rs* = 0.006; *p* = 0.9966), LDL (*rs* = 0.0260; *p* = 0.8420), and VLDL (*rs* = 0.022; *p* = 0.8658).

## 4. Discussion

Abnormalities in lipid and lipoprotein levels are commonly reported in physiological and nonphysiological conditions [8, 16]. The changes that occur during inflammation and infection are part of the innate immune response and therefore are likely to play an important role in protecting the host [8, 9, 16, 21, 22]. In malaria, the exact mechanism is unclear, but it is likely the enrollment of factors of* Plasmodium*, human host, or both [10, 13, 21]. Lipids play a crucial role in the metabolism of* Plasmodium* in both phases of its life cycle in the human host [10]. These organisms use cholesterol and phospholipids from the host to their metabolic requirements, such as membrane or haemozoin formation [23–25]. Additionally, it is likely that the parasite modifies metabolic pathways of lipids in the hepatocytes. Finally, oxidative stress has been associated with oxidation of lipoproteins, contributing to the abnormalities of lipids levels [17, 24, 26].

In the study, all participants have total cholesterol levels within normal range values. However, such levels were significantly lower in children with malaria compared to the healthy control. Reduction of total cholesterol level is a common finding in patients with malaria and has been associated with metabolic requirements of* Plasmodium* for their optimal development in hepatocytes, since there is no biological pathway for sterol production, which is obtained from the exogenous or endogenous pathways of the human host [10, 11, 23, 27].

Cholesterol rich lipoprotein levels, such as LDL and HDL, have also decreased significantly in children with malaria at admission, compared to healthy children. These changes agree with most studies designated to evaluate lipid abnormalities in patients with malaria and other infectious diseases [9, 11, 16, 28]. For instance, a previous study with Nigerian children with falciparum malaria has shown a significant decrease of total cholesterol and cholesterol rich lipoproteins in complicated cases, but, in uncomplicated cases, only the LDL fraction was significantly lower in children when compared to the control group [29]. Another study in the same country has shown a similar pattern of lipid changes compared to the current study [30]. In fact, a high proportion of children with malaria present HDL and LDL levels below normal range. The mechanisms by which inflammation and infection decrease HDL levels are uncertain, but in acute malaria two factors contribute for these findings: the redox imbalance which favors the oxidation of lipoproteins; the mobilization of cholesterol internalized by lipoproteins by* Plasmodium* [17, 23, 26]. Additionally, the occurrence of functional alterations of these lipoproteins is likely, as seen in other inflammatory disorders, such as the diminished ability of HDL to prevent the oxidation of LDL [8].

On the other hand, triglycerides and VLDL, a lipoprotein rich in triglycerides, increased significantly in children with malaria, when compared to the control. A plausible explanation is an increase in the hepatic VLDL production and secretion in serum accomplished by a decrease in the clearance of this lipoprotein. Additionally, the role of cholesterol ester transfer protein in mediating the exchange of triglycerides from triglyceride rich lipoproteins to other lipoproteins is likely [16, 26].

The severity of infection was associated with abnormalities in lipid levels in different studies, but the results were inconclusive [10, 31]. Some authors have found a significant relation between parasite count and lipid levels, which is more evident in patients with high level of parasitaemia and in complicated cases [27, 32]. In the study, there was a lack of relation between parasite count and lipid levels at admission, which may be due to the low level of parasitaemia and the mild signs and symptoms of the disease in children enrolled in the study [27].

There is a continuous exposure to the* Anopheles* bites in this endemic setting and most participants of both groups have a history of malaria. Thus, it may be worth assessing if the history of malaria may influence lipid levels. In the study, the number of previous episodes of malaria was not associated with the lipid levels. A plausible explanation for such finding is the temporality of changes in lipid profiles. Studies evaluating temporal changes in lipid profiles of patients with malaria have demonstrated inconclusive results, ranging from a few days to one year [11, 14, 32]. In the study, the temporal evaluation of lipid levels showed a rapid alteration, with the values of all lipid parameters on D14 similar to the ones for the control group. These changes may be related to both the clearance of parasites and the concurrent improvement of nutritional status of patients in the convalescence period due to the normal ingestion of food after cessation of signs and symptoms of the infection such as the fever. The cessation of fever and the lipids liberated from parasite destruction may also influence the changes in lipid levels [9, 10, 23]. Finally, the profiles of temporal changes of lipid levels seen in the study support the exclusion of patients with history of disease in the previous two months, which can be considered a plausible period for total recovery of the nutritional status and lipid levels of this group population.

Further studies should investigate the impact of low levels of cholesterol rich protein on the development, cognition, and nutritional status of children continuously exposed to malaria as well as the atherosclerosis risk and related diseases in such patients.

## 5. Conclusion

Changes in the lipid profile are significant in children with low level of parasitaemia by* P. vivax* and mild signs and symptoms. Lipid abnormalities were characterized by decreased levels of total cholesterol, LDL, and HDL and by the increased levels of VLDL and triglycerides. These changes were transient and were not associated with parasite count, as well as with the history of malaria of patients.

## Figures and Tables

**Table 1:** Baseline characteristics of participants.

Variable	Cases (*n* = 43)	Control (*n* = 32)	*p*
Age, years	7.5 (2)	5.5 (3)	0.5221
Parasitaemia at admission, geometric average	3347	—	
Previous episodes of disease, number of patients			
0	12	7	0.8024
Once, twice, or 3 times	10	9	
4 times or more	21	16	
Parasite clearance, hours	36 (12)	—	
Fever clearance, hours	24 (12)	—	
Hematocrit, (%)	36 (2)	37 (2)	0.5142
Hemoglobin, g/dl	12 (3)	13 (4)	0.6198
